# A Case of Biatrial-dependent Tachycardia

**DOI:** 10.19102/icrm.2021.120206

**Published:** 2021-02-15

**Authors:** Khalil Kanjwal, Shakeel Jamal, Abdul Q. Haji

**Affiliations:** ^1^McLaren Greater Lansing Hospital, Lansing, MI, USA; ^2^Department of Medicine, Central Michigan University, Saginaw, MI, USA; ^3^Martinsburg VA Medical Center, Martinsburg, WV, USA

**Keywords:** Ablation, atrial fibrillation, atrial flutter, Bachman’s bundle, mapping

## Abstract

We present an interesting case of atrial flutter in a patient with previous pulmonary vein isolation. The entirety of the atrial flutter cycle length was mapped to the left atrium; however, an atrial flutter could not be terminated from the left side. Subsequently, the right atrium was mapped and an area of earliest activation was noted in the junction between the superior vena cava and right atrium. Ablation performed in this area terminated the flutter. We believe that both the left atrium and the portion of the right atrium indicated were part of the circuit and herein discuss the likely mechanism of the biatrial dependence of this tachycardia.

## Case presentation

**Figure 1:**
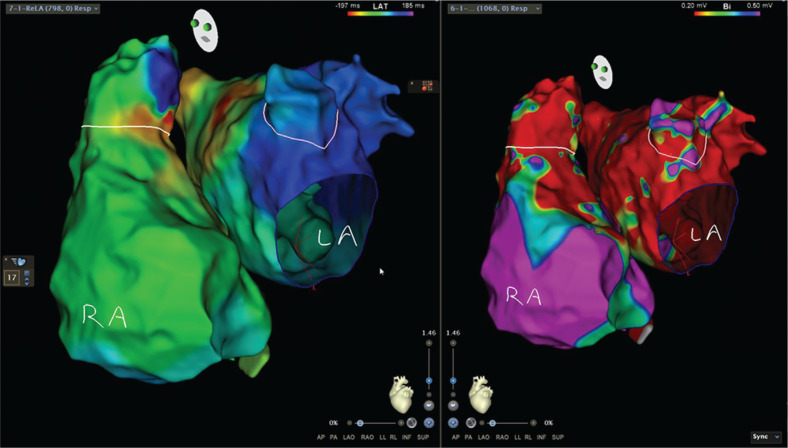
Simultaneous LA and RA activation maps showing an area of earliest activation in the roof in the LA and RA–SVC junction. The voltage map shows extensive scarring in the LA and near the SVC–RA junction. More than 90% of the TCL was mapped in the LA. LA: left atrium; RA, right atrium; SVC: superior vena cava.

**Figure 2:**
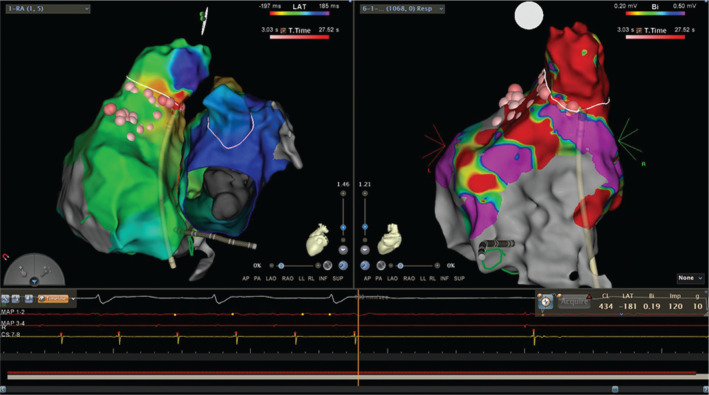
Ablating in the area of the SVC and right atrial junction resulted in termination of the flutter, proving that the RA was part of the circuit. RA: right atrium; SVC: superior vena cava.

A 73-year-old man with a history of symptomatic persistent atrial fibrillation (AF), coronary artery bypass graft, hypertension, and diabetes mellitus who had previously undergone AF radiofrequency ablation with pulmonary vein isolation (PVI) and roof, mitral isthmus, and cavotricuspid isthmus ablation presented with atrial flutter with proximal to distal activation in the coronary sinus. Given his prior extensive left atrial ablation, it was decided to map the left atrium (LA) first. After transeptal access, extensive fast anatomical, voltage, and activation mapping were performed. All veins were isolated. Activation mapping revealed that the whole tachycardia cycle length (TCL) was mapped in the LA. Also, there was an area in the roof near the right superior pulmonary vein (RSPV) anteriorly that showed a gap in the previous roof ablation line **(Figure 1 and Video 1)**. Entrainment in this area resulted in a close postpacing interval (PPI) suggestive of the area being part of the circuit. Entrainment also revealed that the cavotricuspid isthmus and the lateral right atrium (RA) were not part of the circuit. Ablation performed in this area prolonged the TCL without terminating the tachycardia and no change in activation was noted. Subsequently, we decided to map the RA, so activation, fast anatomical, and voltage mapping of the RA were performed. There was an area of early activation near the junction of the superior vena cava (SVC) and the RA and some scarring was noted in this area on the voltage map. This area was anteriorly adjacent to the RSPV roof. Entrainment from this area also resulted in close PPI again, suggestive of the area being part of the circuit. Ablation was finally performed near the RA–SVC junction, resulting in TCL prolongation and tachycardia termination **(Figure 2)**.

## Discussion

PVI has emerged as the most effective rhythm-control strategy. Common problems that may arise following PVI, however, include both focal and macro-reentrant atrial tachyarrhythmias. The ablation of these tachyarrhythmias is often challenging. Our patient underwent extensive LA ablation and subsequently presented with a highly symptomatic recurrent atrial flutter. Post-PVI atrial flutters usually originate in the LA and it is common practice to map the LA initially. Our patient underwent detailed LA activation mapping. The whole TCL was mapped in the LA and, therefore, it was deemed likely that the LA was part of the tachycardia circuit. However, attempts at ablation in the LA in the areas where there was a gap in the previous ablation line near the RSPV and roof failed to terminate the tachycardia. It was only further extensive mapping and ablation near the SVC–RA junction, adjacent to the ablation sites in the LA, that the flutter was terminated. Successful termination of the flutter while ablating in the RA proved that the RA was part of the tachycardia circuit. Moreover, the fact that the whole TCL was mapped to the LA and the termination of the tachycardia was achieved by ablating at the SVC–RA junction makes it highly likely that both the LA and the RA were part of the tachycardia circuit.

Predisposing factors for biatrial tachycardia include the resumption of conduction through a previously performed ablation line (residual reconnection gaps along the prior encircling ablation lines around pulmonary veins), preferential conduction through Bachmann’s bundle, and prior surgery involving the atria such as atrial septal defect closure or the maze procedure.^1,2^ Of these, preferential conduction through Bachmann’s bundle has been proposed as a predisposing factor for atrial tachycardia development and it is debatable whether this is related to the presence of specialized conduction tissue or because of the anisotropic orientation of the muscle.^1,2^ Residual reconnection gaps have been extensively characterized and studied.^3^ Biatrial tachyarrhythmias have been reported previously as well.^4–6^

A case of biatrial Bachmann’s bundle–dependent tachycardia was previously discussed by Gracia et al.,^4^ who reported a biatrial flutter in a patient with prior cardiac surgery and the anomalous insertion of Bachmann’s bundle in the SVC. They were also able to terminate the tachycardia by isolating the SVC.

While it is possible that the present tachycardia was Bachman’s bundle–dependent other possibilities need to be entertained as well. For example, it is also possible that interatrial tissue was part of the circuit and required ablation to be performed on both sides or a residual conduction gap was left in the roof of the LA and the prior ablation lesion was not deep enough and necessitated additional ablation from the right atrial side to seal the gap. Regardless of the mechanism of this biatrial tachycardia, it is very important for the electrophysiologist to have a low threshold of mapping in the RA when a seemingly left-sided flutter cannot be terminated by extensive mapping and ablation in the LA.

## Conclusion

In patients with post-PVI atrial flutter that is difficult to ablate, RA mapping should be performed as it may reveal an alternative approach to a successful ablation.

## Figures and Tables

**Video 1:** Propagation map showing simultaneous activation from the adjacent areas of both the LA and RA.
